# Using trend arrows in continuous glucose monitoring systems for insulin adjustment in clinical practice: Brazilian Diabetes Society Position Statement

**DOI:** 10.1186/s13098-020-00607-2

**Published:** 2021-01-03

**Authors:** M. Rodacki, L. E. Calliari, A. C. Ramalho, A. G. D. Vianna, D. R. Franco, K. F. S. Melo, L. R. Araujo, M. Krakauer, M. Scharf, W. Minicucci, R. Ziegler, M. Gabbay

**Affiliations:** 1 Department of Internal Medicine, Federal University of Rio de Janeiro (UFRJ), Rio de Janeiro, RJ Brazil; 2 Pediatric Endocrinology Unit, Pediatric Department, Santa Casa de São Paulo School of Mediccal Sciences, São Paulo, Brazil; 3 Department of Endocrinology, Federal University of Bahia, Salvador, BA Brazil; 4 Curitiba Diabetes Center, Hospital Nossa Senhora das Graças, Curitiba, PR Brazil; 5 CPCLIN/DASA Clinical Research Center, São Paulo, Brazil; 6 Diabetes Secion, Hospital das Clinicas, University of São Paulo (USP), Quasar Telemedicine (Glic), São Paulo, Brazil; 7 Endocrinology Section, School of Medical Sciences, Belo Horizonte, MG Brazil; 8 Diabetes and Endocrinology, Science Valley Research Institute, Santo André, SP Brazil; 9 Endocrinology Section, University of Campinas (UNICAMP), Campinas, SP Brazil; 10 Diabetes Clinic for Children and Adolescents, Munster, Germany; 11 Diabetes Centre-UNIFESP, Federal University of São Paulo, São Paulo, Brazil

**Keywords:** Trend arrows, CGM, Diabetes mellitus, Insulin

## Abstract

This manuscript reports the Brazilian Diabetes Society Position Statement for insulin adjustments based on trend arrows observed in continuous glucose monitoring systems. The Brazilian Diabetes Society supports the utilization of trend arrows for insulin dose adjustments in patients with diabetes on basal-bolus insulin therapy, both with multiple daily insulin doses or insulin pumps without closed-loop features. For those on insulin pumps with predictive low-glucose suspend feature, we suggest that only upward trend arrows should be used for adjustments. In this paper, tables for insulin adjustment based on sensitivity factors are provided and strategies to optimize the use of trend arrows in clinical practice are discussed.

## Background

Continuous glucose monitoring (CGM) brought important advances to diabetes care. One of the important advantages of CGM over self-monitoring blood glucose alone is the presence of trend arrows. These arrows are based on interstitial glucose variation over the previous 15 min and allow estimation of the rate of glucose rise or decline over the next 30 to 60 min [1]. The underlying definition of an arrow varies according to the CGM manufacturer (Table 1).

**Table 1:** Predicted glucose variation (in mg/dL) according to trend arrows in 30 min

Arrow	Abbott Freestyle/Libre–Senseonics/Eversense	Medtronic Veo	Medtronic 640G	Dexcom
↑↑↑	NA	NA	+ > 90	NA
↑↑	NA	+ > 60	+ 60 to 90	+ > 90
↑	+ > 60	+ 30 to 60	+ 30 to 60	+ 60 to 90
	+ 30 to 60	NA	NA	+ 30 to 60
→	+ < 30	NA	NA	± < 30
	− 30 to 60	NA	NA	− 30 to 60
↓	− > 60	− 30 to 60	− 30 to 60	− 60 to 90
↓↓	NA	− > 60	− 60 to 90	− > 90
↓↓↓	NA	NA	− > 90	NA

NA: non-applicable

The usage of trend arrows for bolus insulin dose adjustments has been suggested by several authors. This position statement expresses the opinion of Brazilian experts on the use of trend arrows for this purpose. The incorporation of trend arrows would add one more component to the insulin bolus calculation, as follows: (1) Meal bolus: insulin dose calculated to “cover” food according to carbohydrate-to-insulin ratio; (2) Correction bolus: supplemental insulin dose to correct hyperglycaemia, calculated according to the sensitivity factor (SF) and target glycemia; (3) Dose adjustment according to trend arrows. If there is a trend arrow pointing upwards, an increase in bolus insulin dose should be performed. Otherwise, in the presence of a downward arrow, a bolus reduction or other preventive measure may be taken to reduce the risk of hypoglycaemia, such as carbohydrate intake or stop the infusion of an insulin pump.

Change in bolus insulin dose according to trend arrows has been suggested for both patients with multiple daily injections and insulin pumps [2]. For users of insulin pump with the predictive low-glucose suspend (PLGS) feature, only the arrows for management of hyperglycemia (upward arrows) should be used for insulin adjustments, as the insulin pump algorithm has incorporated the downward arrows for hypoglycemia prevention [3]. For the newer insulin pumps with automatic basal and/or bolus adjustments (hybrid closed loop or full closed loop systems), it is reasonable to avoid insulin dose adjustments based on trend arrows, as algorithms are designed to automatically correct oscillations without external interference. However, even in these cases (insulin pumps with automatic basal) patients can still use the arrows to evaluate trends after meals as well as to help to determine the carbohydrate amount to be used in order to avoid hypoglycemic episodes.

The use of trend arrows for bolus insulin adjustments was firstly suggested by the DirecNet Applied Treatment Algorithm (DATA) study with Freestyle Navigator™ real-time continuous glucose monitor, which indicated an increase of bolus insulin dose by 10% or 20% for one or two arrows in the upward direction and a reduction in the same proportion for one and two downward arrows, respectively [4]. Pettus, Edelman and Scheiner suggested a different approach for the use of trend arrows in clinical practice [5, 6]. The arrows allow the estimation of glucose changes for the next 15 to 30 min. Therefore, they recommend adding or subtracting the predicted change in glucose level from the actual glucose measurement obtained by CGM. Hence, the insulin bolus is calculated based on this new corrected value (Table 2).

**Table 2:** Previous insulin dose adjustments based on trend arrows recommendations

Arrow			JDRF[4]	Scheiner [6]	Pettus/Edelman [5]	Klonoff/Kerr [10]	Aleppo [7]				Laffel [9]	
Abbott	Dexcom	Medtronic										
							Pre-meal		2–4 h Post meal			
							SF	Change	Glucose	Change	SF	Change
	↑↑	↑↑↑	+ 20%	+ 50 mg/dL	+ 100 mg/dL	+ 2 U	< 25	+ 4.5 U			< 25	+ 4.0 U
							25–< 50	+ 3.5 U			25– 50	+ 3.0 U
							50–< 75	+ 2.5 U	> 250 mg/dL	Correction acording to SF; check ketones	50– < 75	+ 2.0 U
							≥ 75	+ 1.5 U			75– < 125	+ 1.0 U
											≥ 125	+ 0.5 U
↑	↑	↑↑	+ 20%	+ 25 mg/dL	+ 75 mg/dL	+1,5 U	< 25	+ 3.5 U			< 25	+ 3.0 U
							25– < 50	+ 2.5 U	150–250 mg/dL	Observe, consider correction according to SF	25– < 50	+ 2.0 U
							50– < 75	+ 1.5 U			50– < 75	+ 1.0 U
							≥75	+1.0 U			75– < 125	+ 0.5 U
											≥ 125	+ 0 U
↗	↗	↑	+ 10%	+ 0	+ 50 mg/dL	+ 1 U	< 25	+ 2.5 U	No adjustments		< 25	+ 2.0 U
							25–< 50	+ 1.5 U			25– < 50	+ 1.0 U
							50–< 75	+ 1.0 U			50– < 75	+ 0.5 U
							≥ 75	+ 0.5 U			75– < 125	+ 0 U
											≥ 125	+0 U
→	→		+ 0	+ 0	+ 0	+ 0	No adjustments		No adjustments		No adjustments	
↘	↘	↓	− 10%	− 0	− 50 mg/dL	− 1 U	< 25	− 2.5 U	Glucose	Change	< 25	− 2.0 U
							25– < 50	− 1.5 U	Near 100 mg/dL	Consider 15gCHO Test 20 min	25– < 50	− 1.0 U
							50– > 75	− 1.0 U			50- < 75	− 0.5 U
							≥ 75	− 0.5 U	Near 150 mg/dL	Test 30 min	75- < 125	− 0 U
											≥125	− 0 U
↓	↓	↓↓	− 20%	− 25 mg/dL	− 75 mg/dL	− 1.5 U	< 25	− 3.5 U	Near 100 mg/dL	Consider 15gCHO Test 20 min	<25	− 3.0 U
							25– < 50	− 2.5 U			25– < 50	− 2.0 U
							50– < 75	− 1.5 U	Near 150 mg/dL	Test 15 min	50– < 75	− 1.0 U
							≥ 75	− 1.0 U			75– < 125	− 0.5 U
											≥125	− 0 U
	↓↓	↓↓↓	− 20%	− 50 mg/dL	− 100 mg/dL	− 2 U	< 25	− 4;5 U	Near 100 mg/dL	30 g fast-acting carbohydrate	<25	− 4.0 U
							25– < 50	− 3.5 U			25- < 50	− 3.0 U
							50– < 75	− 2.5 U			50- < 75	− 2.0 U
							≥75	− 1.0 U			75- < 125	-− 1.0 U
									Near 150 mg/dL	Test 15 min	≥125	− 0.5 U

The algorithm suggested by Kudva et al. [8] is similar to the one suggested by Aleppo et al. [7], except that the range 150–250 mg/dL for post-meal adjustments (for upward arrows) should be replaced by 180-250 mg/dL. For other glucose values no adjustments are suggested by Aleppo or Kudva [7, 8]. The recommendations suggested by Laffel et al. [9] and JDRF [4] were elaborated for children and adolescents

Other authors have recognized the importance of simplifying these recommendations by suggesting the addition or reduction of absolute insulin units in the calculation rather than the percentage, as shown in Table 2. However, this strategy does not consider each individual’s sensitivity to insulin. Subsequently, categorization of insulin dose adjustments according to each patient’s SF was also proposed, considering individual variations in insulin sensitivity and the necessity to provide different dose adjustments based on that. Aleppo et al. proposed different adjustments for pre and postprandial period, using the sensitivity factor for preprandial adjustments and glucose level at the time of the test for postprandial adjustments (2–4 h after meal) for Dexcom sensor for adults [7], which was corroborated by Kudva [8] et al. for Freestyle Libre™ sensor (Table 2). Laffel et al. proposed a similar dose adjustment for kids using Dexcom™, with slight modifications: (1) using − 0.5 units less in all proposed changes; (2) incorporation of recommendations for SF ≥ 125 mg/dL (as often used in children); (3) use of trend arrows for bolus insulin dose adjustments only pre-meal or ≥ 3 h after a meal, using the pre-meal algorithm. Earlier post-meal adjustments were not recommended for the pediatric population by Laffel et al. [9].

More recently, Ziegler et al. proposed bolus insulin dose adjustments based on trend arrows considering current glucose and sensitivity factor at all times without differentiating pre and postprandial adjustments for all sensors [10]. In most situations, this method uses approximately the doses proposed by Aleppo et al. for level 1 hyperglycemia (180–250 mg/dL), milder changes for glucose levels within the target range (70–180 mg/dL) and more significant changes for patients with level 2 hyperglycemia (> 250 mg/dL). This strategy allows for a more conservative approach for glucose values within the target range, which may reduce the risk of hypoglycemia. On the other hand, it acts more aggressively in the presence of severe hyperglycemia, which is acutely associated with greater insulin resistance. Tables with suggested bolus insulin dose adjustments based on trend arrows, insulin sensitivity and glucose levels were elaborated for use in clinical practice. Ziegler proposed different dose adjustments for adults with type 1 diabetes (Table 3), children with T1D and individuals with type 2 diabetes (T2D). In general, recommendations for kids include 0.5 units of insulin less for each recommended adjustment, when compared to the algorithm proposed for T1D, as well as the inclusion of additional SF categories: 75–124, 125–199 and ≥ 200 mg/dL. Recommendations for patients with T2D include, in general, addition of 0.5 units of insulin for each adjustment compared to adult T1D [10].

**Table 3:** Insulin dose adjustments based on trend arrows recommendations for adults with T1DM proposed by Ziegler et al

Rate if change			Glucose levels												
			< 70	70–180				180–250				>250			
				Correction factor											
Abbott/Roche	Dexcom	Medtronic		< 25	25– < 50	50– < 75	> 75	< 25	25– < 50	50– < 75	> 75	< 25	25– < 50	50– < 75	> 75
				Recommended changes											
	↑↑	↑↑↑	CU	+ 3.5 U	+ 2.5 U	+ 1.5 U	+ 1 U	+ 4.5 U	+ 3.5 U	+ 2.5 U	+ 1.5 U	+ 5 U	+ 4 U	+ 3 U	+ 2 U
↑	↑	↑↑		+ 2.5 U	+ 2 U	+ 1 U	+ 0.5 U	+ 3.5 U	+ 2.5 U	+ 1.5 U	+ 1 U	+ 4 U	+ 3 U	+ 2 U	+ 1.5 U
↗	↗	↑		+ 1.5 U	+ 1 U	+ 0.5 U	+ 0 U	+ 2.5 U	+ 1.5 U	+ 1 U	+ 0.5 U	+ 3 U	+ 2 U	+ 1.5 U	+ 1 U
→	→		1 fast-acting CU	+ 0 U	+ 0 U	+ 0 U	+ 0 U	+ 0 U	+ 0 U	+ 0 U	+ 0 U	+ 0 U	+ 0 U	+ 0 U	+ 0 U
↘	↘	↓	2 fast-acting CU	− 2.5 U	− 1.5 U	− 1 U	− 0.5 U	− 2 U	− 1 U	− 0.5 U	− 0.5 U	− 2.5 U	− 1 U	− 0.5 U	− 0 U
↓	↓	↓↓		− 3.5 U	− 2.5 U	− 1.5 U	− 1 U	− 3 U	− 2 U	− 1 U	− 1 U	− 3.5 U	− 2 U	− 1 U	− 0.5 U
	↓↓	↓↓↓		− 4.5 U	− 3.5 U	− 2.5 U	− 1.5 U	− 4 U	− 3 U	− 1.5 U	− 1.5 U	− 4 U	− 2.5 U	− 1 U	− 0.5 U

Adapted from Ziegler et al. [10]. For individuals with T2D, 0.5 units should be added. For children, 0.5 units should be reduced. Recommendations for SF between 125 and 200 mg/dL and > 200 mg/dL are provided for children [10]

Regardless of the chosen algorithm for bolus insulin dose adjustment based on trend arrows, the time of the last meal and the last bolus administration should be considered, to estimate the proportion of circulating active insulin. There is a significant effect of active insulin up to 2 h after administration of an insulin bolus [11]. Therefore, in this period, when identifying hyperglycemia, even with upward arrow, the best approach is usually careful observation of the CGM values, without immediate administration of supplemental insulin dose. If glucose values are still rising 2 h after the meal bolus, insulin can be given accordingly.

Other factors that may influence glucose levels should also be considered when adjusting insulin based on trend arrows, such as exercise, stress, menstruation, sick days or use of medications that cause hyperglycemia (e.g.: corticosteroids) [10]. This could mean that even more insulin is needed to correct hyperglycemia. Use of trend arrows during exercise must be personalized to each individual, based on the exercise type, intensity and duration, in addition to prior experience with managing glucose levels during exercise [12]. We also recommend using the downward arrows to help prevent hypoglycemia during exercise and avoid administering extra insulin based on upward trend arrows during high intensity exercise.”

## Limitations of trend arrows

The use of trend arrows by patients for decision making in real time has some limitations. Firstly, the trend arrows are based on retrospective data collected by a glucose sensor. For example, there may be cases where the arrow based on retrospective measurements points downwards, but the glucose level has already stabilized. This phenomenon may not be detected by the trend arrows but may be visualized by looking at the glucose level curve of the last hour, which is displayed on the device. In these situations, decisions must be made carefully. Considering the average time interval between the sensor and the YSI reference values (4.5 ± 4.8 min) and the performance and usability of a factory-calibrated Flash Glucose Monitoring System, we recommend that the patient should observe the trend arrow and analyze the graphical glycemic curve together, considering the last 10 min of the curve, to minimize the chance of error caused by this phenomenon [13, 14].

Moreover, clinical studies are still required to investigate if the use of trend arrows to modify insulin dose for patients with diabetes result in improvement of HbA1c, glucose variability, time in glucose range or hypoglycaemic events.

## The Brazilian Diabetes Society recommendations for trend arrows use in bolus insulin dose adjustments

As it is important to adapt the use of trend arrows to each unique regional context, the Brazilian Diabetes Society elaborated recommendations for their rationale use in the bolus insulin dose calculation. Firstly, the Brazilian Diabetes Society (SBD) supports the utilization of trend arrows for bolus insulin dose adjustments in patients with diabetes that use basal-bolus insulin therapy. The strategy seems reasonable for patients with multiple daily doses of insulin injections and pumps without automatic dose adjustments. As the algorithm created by Ziegler et al. suggests changes according to sensitivity factor and glucose levels at all times, we support the use of this strategy, although it is important to emphasize that clinical studies have not been performed to validate either of these algorithms or to determine if one is superior to the other [10].

Could European recommendations be extrapolated for other populations? In Brazil, a current problem is the misuse of basal insulin in higher than recommended doses for patients on basal-bolus regimen [15]. This has been associated with worse glycemic control, increased risk of hypoglycemia and post-prandial hyperglycemia [16]. Medical education strategies have been pursued to avoid this excessive use of basal insulin. However, a major concern of SBD was that the use of trend arrows could lead to the risk of increasing the number hypoglycemia episodes in individuals on higher basal insulin doses. Therefore, SBD recommends that the proportion of basal insulin in basal bolus insulin therapy should be ≤ 50% and that trend arrows insulin dose adjustments should not be performed for those with basal insulin proportions > 50%. We also recommend that these dose adjustments should be performed for patients that use short-acting insulin analogs preferably 15 min or more before meals or at meals for those who use fast rapid insulin (FIASP), which is indicated for patients with type 1 DM and patients with type 2 DM in basal bolus insulin therapy.

Another important point is how to simplify the use of the dose adjustments algorithms in clinical practice worldwide. The use of apps seems the ideal strategy to help patients to incorporate changes in the bolus dose calculations. Apps that calculate bolus insulin dose are already available and include the calculation of food bolus and correction bolus, to determine the total bolus amount. The incorporation of trend arrows would represent the addition of a third and feasible step to the process. Analogic solutions may also be used, such as simplified tables that can be provided to patients in the medical or educational appointments. Medical providers may find and provide only the appropriate simple table for each patient according to the SF.

**Fig. 1:**
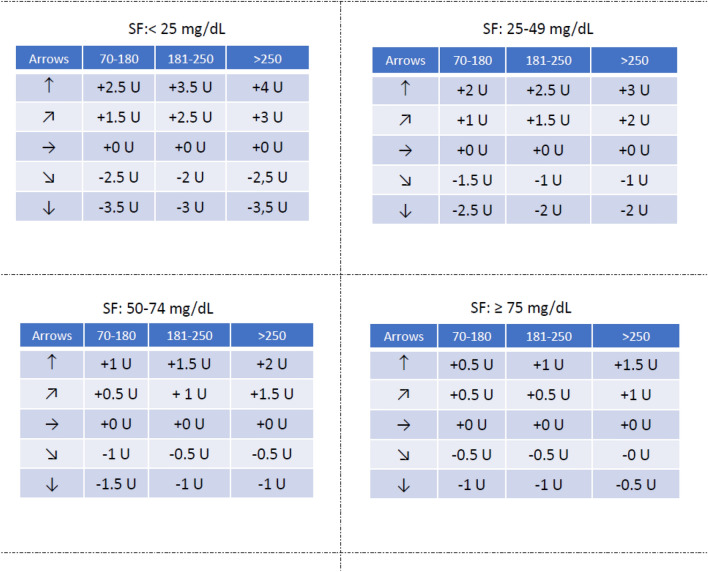
Tables to be provided to adult patients for insulin dose adjustements according to trend arrows (Freestyle Libre Abbott/Roche sensor users)

**Fig. 2:**
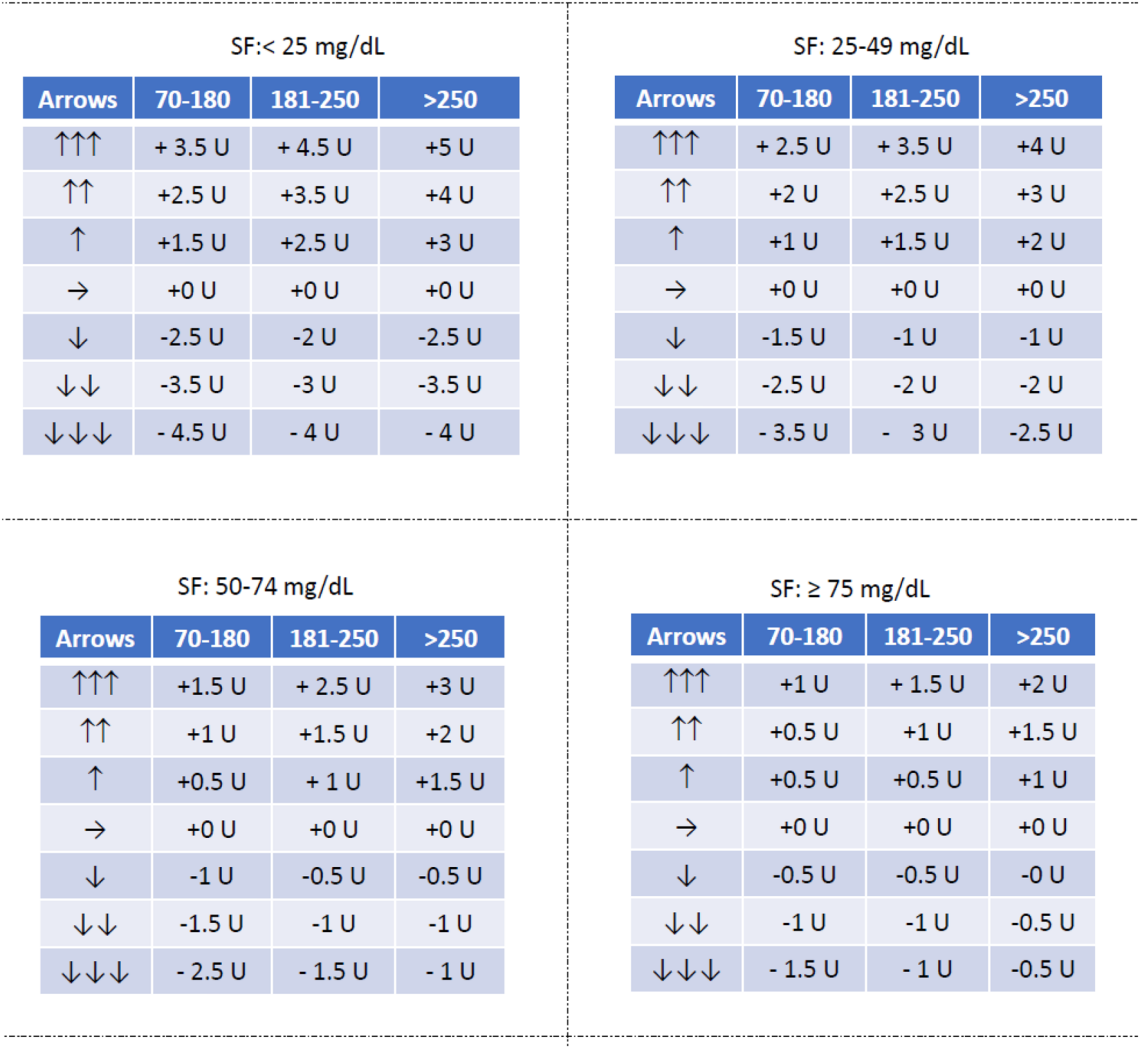
Tables to be provided to adult patients for insulin dose adjustements according to trend arrows (Medtronic sensor users). For patients with PLGS pumps only recommendations for upward arrows should be used. Patients with closed loop systems should not use this algorithm

**Fig. 3:**
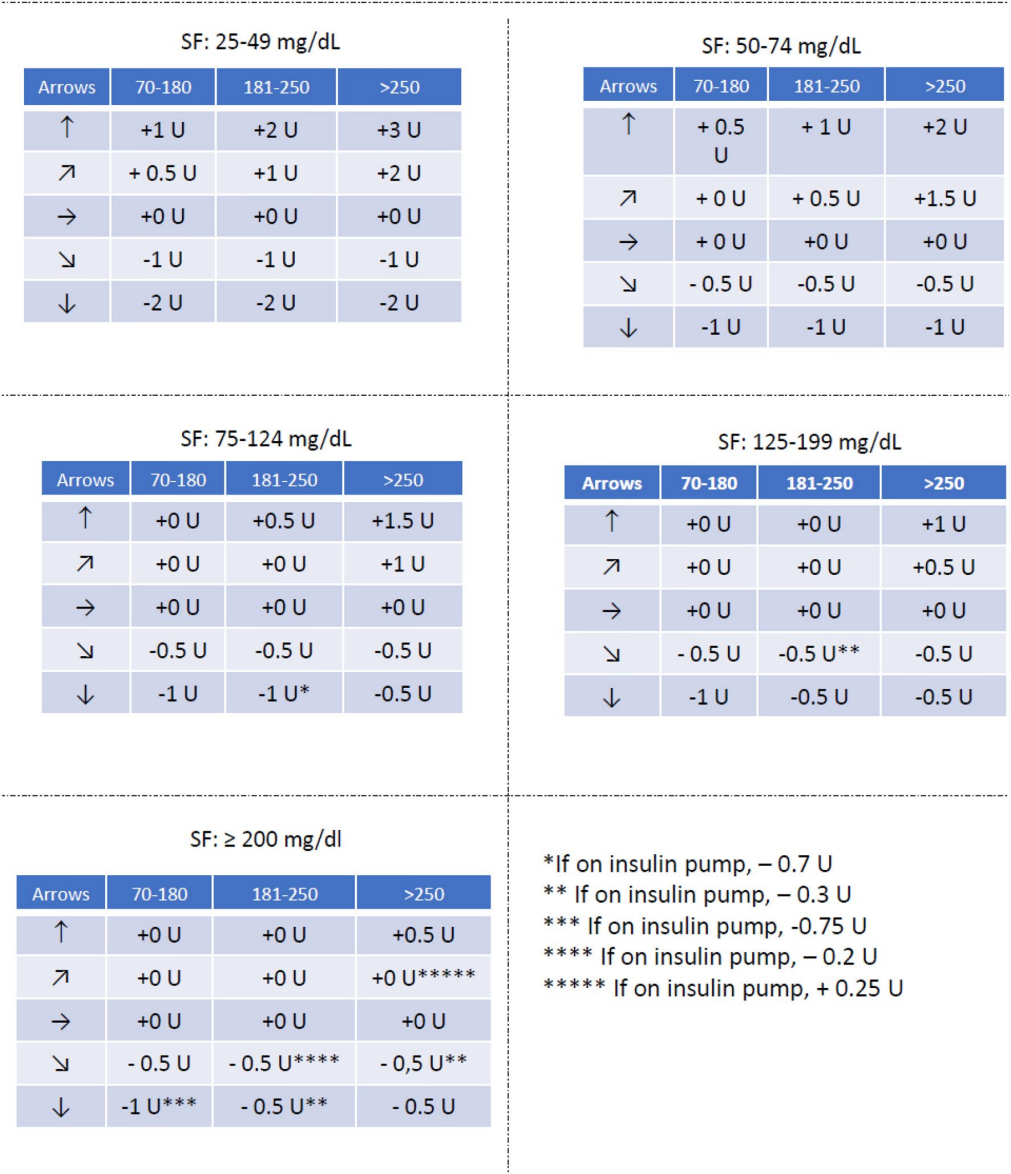
Tables to be provided to children and adolescents for insulin dose adjustements according to trend arrows (Freestyle Libre Abbott/Roche sensor users). *If on insulin pump, − 0.7 U; ** If on insulin pump, − 0.3 U; *** If on insulin pump, − 0.75 U; **** If on insulin pump, − 0.2 U; ***** If on insulin pump, + 0.25 U

**Fig. 4:**
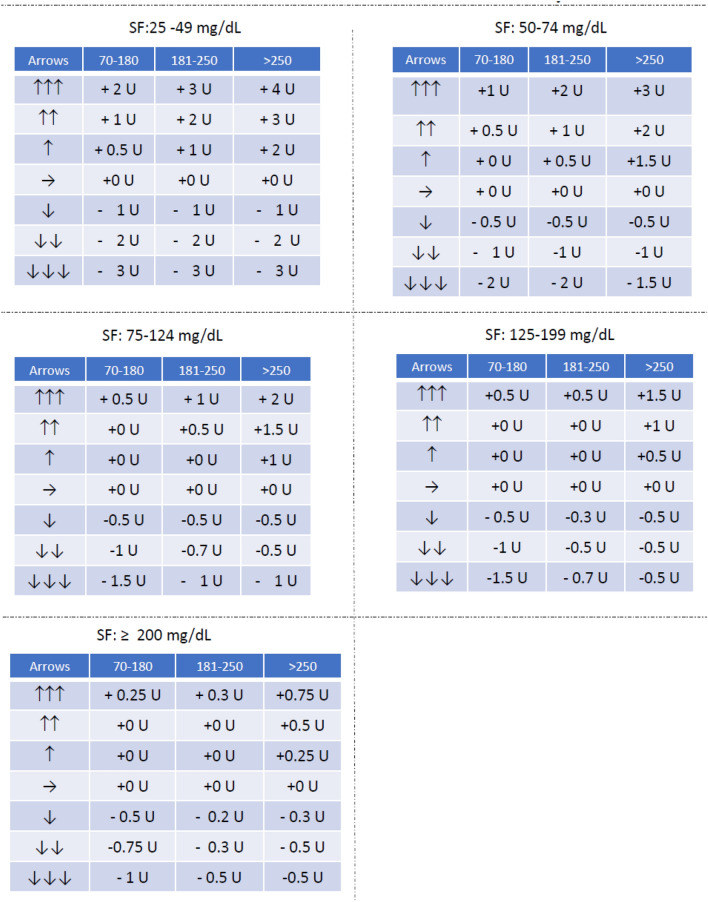
Tables to be provided to children and adolescent patients for insulin dose adjustments according to trend arrows (Medtronic sensor users). For patients with PLGS pumps only recommendations for upward arrows should be used. Patients with closed loop systems should not use this algorithm

In order to simplify clinical care, we suggest the same recommendations for adult patients with type 1 and type 2 diabetes with similar SF in clinical practice (Figs. 1 and 2). Although this may represent a conservative approach for patients with type 2, as doses were established according to type 1 diabetes tables, we believe that this could simplify the use of the method. For patients with type 2 diabetes an SF < 10 mg/dL, more aggressive algorithms might be necessary, as suggested by Ziegler et al. [10]. Figures 3 and 4 indicate the suggested approach for children and adolescents, for different sensors.

A separate table for the use of trend arrows during hypoglycemia was also created (Table 4). The recommendations are based on carbohydrate units (CU). CU is the usual amount of carbohydrates that a person needs to treat or prevent hypoglycemia (15 grams of carbohydrates for most adults but much lower doses might be used for kids).

**Table 4:** Trend arrows use during hypoglycemia

Sensor		Recommendation
Abbott	Medtronic	
↑,	↑↑↑, ↑↑, ↑	1 Carbohydrate unit (CU)
→	→	1 Fast acting CU
, ↓	↓↓↓, ↓↓, ↓	2 Fast acting CU

Carbohydrate unit (quantity of carbohydrates that a patient needs, based on their own experience, to prevent hypoglycemia)

Another concern in using the algorithm proposed by Ziegler et al. in clinical practice is the 0.5 units changes suggested in various cases [10], as most patients in multiple daily insulin injections use pens or syringes with 1 unit scales. Therefore, we suggest approximating the total calculated doses to the nearest whole number (“rounding” the values) for the injection. We suggest approximating the numbers upward during the day and downward at night. In patients at high risk of hypoglycaemia, we suggest that the approximation should be always performed downward.

## Conclusions

In conclusion, this is a SBD position statement based on expert opinions that supports the utilization of trend arrows for dose adjustments in patients with diabetes on basal-bolus insulin therapy, both with multiple daily insulin doses or insulin pumps without closed-loop features. For those on insulin pumps with PLGS feature, we suggest that solely upward trend arrows should be used for adjustments. We recommend the use of dose adjustments suggested by Ziegler et al. [10], that considers SF and glucose measurement at all times. However, we suggest minor modifications aiming to simplify its use. Moreover, we stress that insulin dose adjustments based on trend arrows should be performed only in patients that use basal insulin doses ≤ 50% of the total daily insulin dose and preferentially in those that inject bolus insulin about 15 min before meals for rapid insulin or at meals for ultra-rapid insulin and inhaled insulin.

## Data Availability

Not applicable.
